# Human Wharton’s jelly mesenchymal stem cells protect axotomized rat retinal ganglion cells via secretion of anti-inflammatory and neurotrophic factors

**DOI:** 10.1038/s41598-018-34527-z

**Published:** 2018-11-02

**Authors:** Jose E. Millán-Rivero, Francisco M. Nadal-Nicolás, David García-Bernal, Paloma Sobrado-Calvo, Miguel Blanquer, Jose M. Moraleda, Manuel Vidal-Sanz, Marta Agudo-Barriuso

**Affiliations:** 1 Unidad de Terapia Celular y Trasplante Hematopoyético. Instituto Murciano de Investigación Biosanitaria Virgen de la Arrixaca (IMIB-Arrixaca), Murcia, Spain; 2 Dpto Medicina Interna, Universidad de Murcia, Murcia, Spain; 3 Dpto Oftalmología, Universidad de Murcia, Murcia, Spain; 4 Grupo de Oftalmología Experimental, Instituto Murciano de Investigación Biosanitaria Virgen de la Arrixaca (IMIB-Arrixaca), Murcia, Spain; 5 Present Address: Retinal Neurophysiology Section, National Eye Institute, National Institutes of Health, Bethesda, MD USA

## Abstract

Mesenchymal stem cell (MSC) transplantation is emerging as an ideal tool to restore the wounded central nervous system (CNS). MSCs isolated from extra-embryonic tissues have some advantages compared to MSCs derived from adult ones, such as an improved proliferative capacity, life span, differentiation potential and immunomodulatory properties. In addition, they are more immunoprivileged, reducing the probability of being rejected by the recipient. Umbilical cords (UCs) are a good source of MSCs because they are abundant, safe, non-invasively harvested after birth and, importantly, they are not encumbered with ethical problems. Here we show that the intravitreal transplant of Wharton´s jelly mesenchymal stem cells isolated from three different human UCs (hWJMSCs) delays axotomy-induced retinal ganglion cell (RGC) loss. *In vivo*, hWJMSCs secrete anti-inflammatory molecules and trophic factors, the latter alone may account for the elicited neuroprotection. Interestingly, this expression profile differs between naive and injured retinas, suggesting that the environment in which the hWJMSCs are modulates their secretome. Finally, even though the transplant itself is not toxic for RGCs, it is not innocuous as it triggers a transient but massive infiltration of Iba1^+^cells from the choroid to the retina that alters the retinal structure.

## Introduction

Mammalian central nervous system (CNS) neurons are not replaced upon lesion and death. To date, no therapies are available to stop or at least to delay neuronal degeneration. In the search of neuroprotective therapies, mesenchymal stem cell (MSC) transplantation is emerging as an ideal tool for CNS repair^1,2^ because of their limited antigenicity^3^ anti-inflammatory effects, immunomodulatory properties^4^, and secretion of trophic factors^1,5,6^.

Adult stem cells can be isolated from several sources such as bone marrow (BM), dental pulp, adipose tissue, umbilical cord (UC) or placenta. Extra-embryonic derived MSCs (placenta, UC) have well known advantages with respect to adult-derived ones: they are abundant, safe, non-invasively harvested after birth, and more immunoprivileged, reducing the probability to be rejected by the recipient after their allogeneic/xenogeneic transplantation^7,8^.

UC-derived MSCs are considered one of the major MSCs sources for clinical and research applications. From a practical point of view, the umbilical cord is considered a medical waste tissue and its collection is not related to ethical issues. Besides, it is relatively easy to isolate a great number of MSCs after several passages and broad *ex vivo* expansion. Importantly, compared to MSCs from other sources, UC-MSCs have an improved proliferative capacity, life span, and differentiation potential and show no signs of senescence over serial passages^9^.

MSCs isolated from the human umbilical cord stroma or Wharton’s Jelly (hWJMSC) have been described as the best MSC source among the various compartments of the UC (stroma, veins, arteries, lining, and subamnion). As the other MSC populations from the UC^10^, hWJMSCs retain the same properties throughout the UC length^11^ thus maximising the use of each cord. They offer the best clinical utility as they have less non-stem cell contaminants, can be generated in large numbers with minimal culture, their derivation is quick and easy to standardize, they are rich in stemness characteristics and have high differentiation potential^12^.

Besides de abovementioned advantages, hWJMSCs, have an enhanced expression of neurotrophic factors, and a spontaneous tendency toward a neural lineage differentiation compared to MSCs isolated from adult tissues^13,14^.

A great model to carry out proof of concept assays of *in vivo* neuroprotection on CNS neurons is the axotomy of the optic nerve. The course of retinal ganglion cell (RGC) loss after optic nerve crush (ONC) or transection (ONT) is very well documented: it is first significant, depending on the species (mouse or rat), 3–5 days after the injury and by day 5–7 half of their population is lost. Thereafter, RGC loss slows down (reviewed in^15^). Thus, axotomy-induced RGC death occurs in two phases^16–19^, the first one lasts 9–14 days and causes the loss of ~85% of RGCs. Then RGC death continues slowly and steadily at least up to 15 months after the insult, when ~1% of the original population survives.

Using this model, several works have described the neuroprotection produced by a single administration of trophic factors, such as brain-derived neurotrophic factor (BDNF^20–23^) vascular endothelial growth factor (VEGF^24^), ciliary neurotrophic factor (CNTF^20,25^) or nerve growth factor (NGF^26^).

Likewise, MSC from different sources have been tested on RGC survival after optic nerve damage (bone marrow MSC^6,27–30^ reviewed in^31^; dental pulp stem cells^6^; adipose MSC^6^, and blood stem cells derived from the umbilical cord^32,33^). The observed neuroprotection was associated with the MSC paracrine secretion of diverse trophic factors^6,27–29,33^. In the retina, the neuroprotective potential of hWJMSCs has been studied in retinal degenerations^34^ and ocular hypertension^35^, but not after optic nerve axotomy.

Here we have investigated whether intravitreally administered hWJMSCs neuroprotect axotomized rat RGCs. After characterizing hWJMSCs and assessing their immunomodulatory properties *in vitro*, we asked the following questions: i/are hWJMSCs toxic for RGCs after intravitreal transplant? ii/do hWJMSCs neuroprotect RGCs from axotomy-induced death? for how long?; iii/is there a dose dependent neuroprotection? iv/do hWJMSCs isolated from different umbilical cords induce the same protection? v/do hWJMSCs integrate in the ganglion cell layer? vi/how long do the transplanted cells survive? vii/do these cells express neurotrophic factors/anti-inflammatory mediators after transplant? viii/does this expression evolve with time post-transplant? ix/is the effect the same in injured and intact retinas? and, x/does the xenotransplant affect the rat retinal architecture?

## Results

### Characterization of human Wharton’s jelly mesenchymal stem cells (hWJMSCs)

After isolation, hWJMSCs display a fibroblastic, spindle-shaped morphology similar to MSCs from other sources such as bone marrow, adipose tissue or dental pulp^36–38^. Likewise, hWJMSCs also have an MSC immunophenotypic profile, i.e. they express high levels (>99.5%) of the surface markers CD73, CD90 and CD105, and lack or low expression (<5%) of the hematopoietic markers CD14, CD20, CD34, CD45, the MHC-class II HLA-DR, and the co-stimulatory molecules CD80 and CD86 (Supplementary Fig. 1). However, and in opposition to bone marrow- or adipose-derived MSC, hWJMSCs mostly fail to differentiate toward adipocytes, osteoblasts, and chondroblasts (not shown) as reported^13^.

### Immunological properties of hWJMSCs

**Figure 1:**
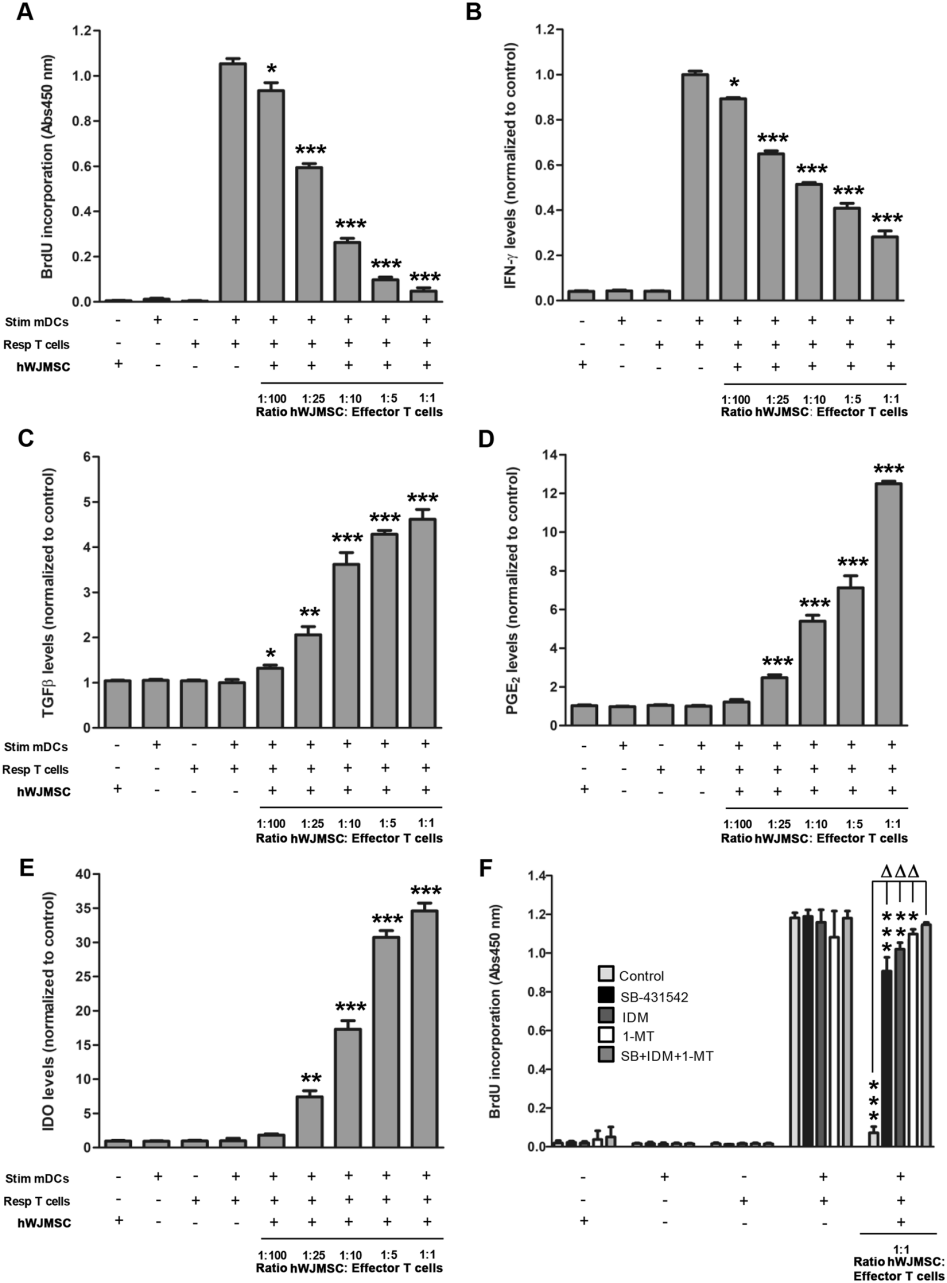
Immunomodulatory properties of human Wharton’s jelly mesenchymal stem cells. (**A**) Column graph showing T cell proliferation (BrdU incorporation assay) after stimulation with allogeneic mDCs. Responder T cell proliferation was significantly inhibited in a dose-dependent manner when hWJMSC were added to the mixed lymphocyte cultures compared to T cell+mDCs without hWJMSC (ANOVA, *p < 0.05 or ***p < 0.001). (**B–E**) Column graphs showing the normalized production of IFN-γ, TGFβ, PGE_2_, and IDO *vs*. control (T cell+mDCs without hWJMSC) in supernatants from the mix lymphocyte cultures in the absence or presence of hWJMSC as before. Compared to control, the production of IFN-γ decreased significantly when hWJMSC were added, while the secretion of TGFβ, PGE_2_, and IDO increased significantly (*p < 0.05, **p < 0.01 or ***p < 0.001). In all cases, this modulation was dependent on the hWJMSC dose. (**F**) Column graph showing T cell proliferation after stimulation with allogeneic mDCs without or with hWJMSC (ratio 1:1), and the absence or presence of the inhibitors SB-431542, IDM or 1-MT. None of the inhibitors alone or in combination influenced the proliferation of T cells in the presence of mDCs. However, the inhibition of T cell proliferation by hWJMSC co-culture was overcome by each inhibitor individually and by their combination (^ΔΔΔ^*p* < 0.001). Full recovery of T cell proliferation was only achieved when the three inhibitors were added at the same time (*p < 0.05, **p < 0.01, or ***p < 0.001, respectively compared to the proliferation of control (T cell+mDCs without hWJMSC). Results are shown as mean ± SD of three independent experiments performed in triplicate according to one-way ANOVA. Abbreviations: Stim mDCs: stimulator mature myeloid dendritic cells, Resp T cells: responder T cells, hWJMSC: human Wharton’s jelly mesenchymal stem cells, IFN-γ: interferon gamma, TGFβ: transforming growth factor beta, PGE_2_: prostaglandin E2, IDO: indoleamine 2,3-dioxygenase, SB-431542: TGFβ inhibitor, IDM: indomethacin (PGE_2_ inhibitor), 1-MT: 1-methyl-tryptophan (IDO inhibitor). n = 3 independent experiments/assay.

First, we studied the capacity of hWJMSCs to suppress the proliferation of T cells stimulated by co-culture with allogeneic myeloid dendritic cells (mDCs), using mixed lymphocyte cultures. In Fig. 1A it is shown that hWJMSCs mediate a dose-dependent inhibitory effect on T cell proliferation compared to T cell proliferation in the absence of hWJMSCs. At the lowest ratio hWJMSCs: effector T cells (1:100), T cell proliferation was significantly reduced compared to the control co-culture in the absence of hWJMSCs (11% reduction, p < 0.05). Furthermore, the addition of a higher number of hWJMSCs to the co-culture led to a more marked inhibition of T cell proliferation, from 44% (ratio 1:25) to 96% (ratio 1:1) (p < 0.001).

Next, we analyzed the effect of hWJMSCs on the production of inflammatory cytokines by stimulated T cells. The co-culture of stimulator mDCs and responder T cells in the presence of hWJMSCs significantly decreased the secretion of IFN-γ (Fig. 1B) and inversely, increased the levels of the anti-inflammatory factors TGFβ, PGE_2_, and IDO (Fig. 1C–E) in a dose-dependent manner which was significant even at the lowest hWJMSCs: effector T cells ratio tested here (1:100) for IFN-γ and TGFβ, and at 1:25 ratio for PGE_2_ and IDO. For all of them the maximum modulation was reached at 1:1 ratio.

### Anti-inflammatory PGE_2_, TGFβ, and IDO play a major role mediating the immunosuppressive effects of hWJMSCs on T cell proliferation

Next, we measured T cell proliferation after mDC stimulation using a 1:1 ratio of hWJMSCs:T cells and different specific inhibitors of the biosynthesis or signaling of TGFβ, PGE_2_ and IDO (SB-431542, indomethacin (IDM) or 1-methyl-tryptophan (1-MT), respectively, Fig. 1F). The addition of SB-431542, IDM, and 1-MT in the absence of hWJMSCs did not affect the maximum proliferative capacity of effector T cells, nor the basal proliferation of stimulator mDCs, responder T cells, or hWJMSCs alone. Again, the proliferation of mDCs-stimulated T cells significantly decreased when hWJMSCs were present in the absence of any inhibitor (94% reduction). However, the addition of any of the inhibitors significantly recovered the proliferative response of T cells (p < 0.001), although this proliferation was still significantly lower than in the absence of hWJMSCs. Remarkably, the simultaneous treatment with the three inhibitors recovered almost entirely the proliferation rate of the T cells, suggesting that PGE_2_, TGFβ, and IDO play a major role mediating the immunosuppressive effects of hWJMSCs on T lymphocytes proliferation after allogeneic stimulation.

### Intravitreally administered hWJMSCs are not toxic for retinal ganglion cells (RGCs)

**Figure 2:**
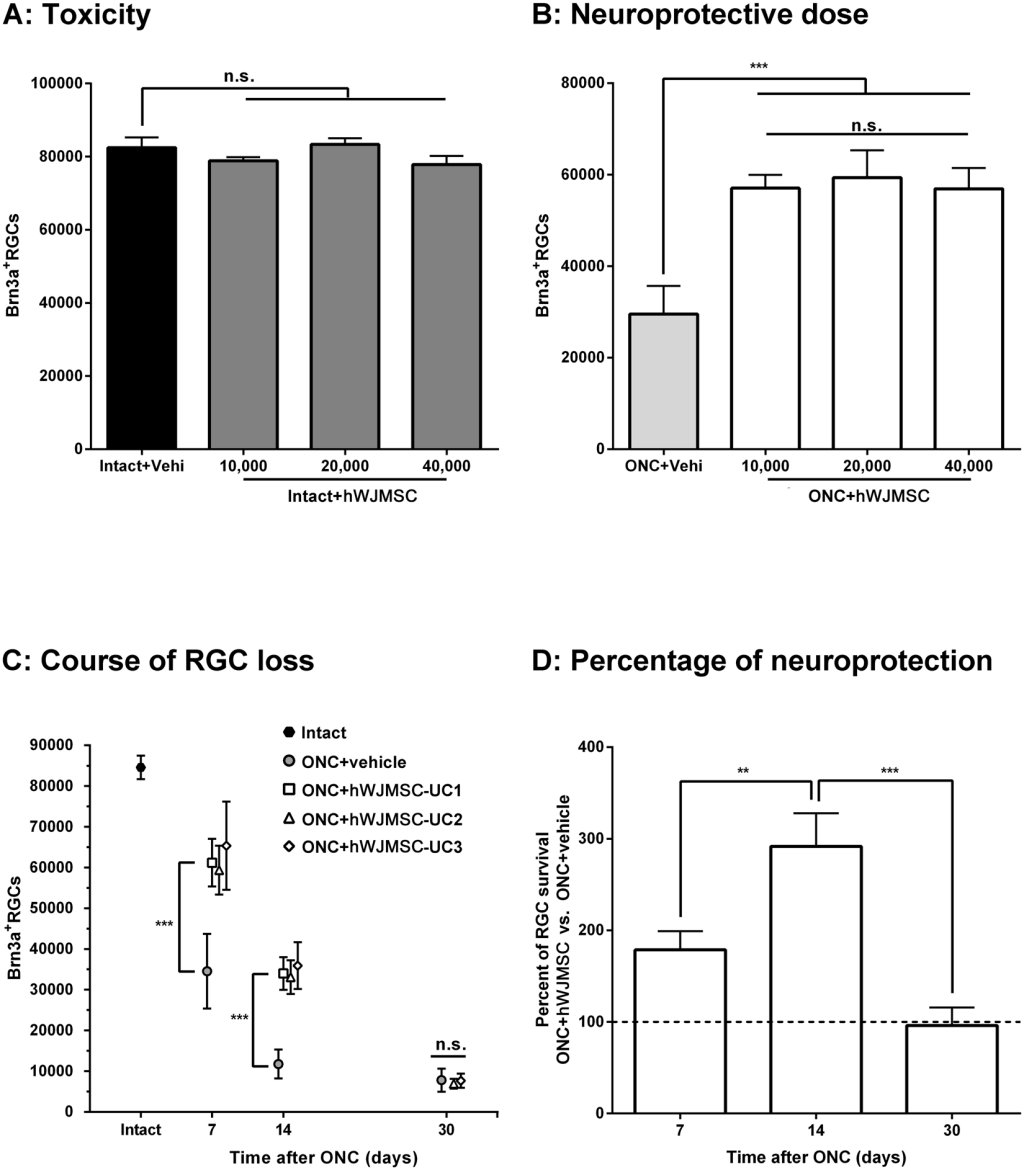
Intravitreal administration of hWJMSC is not toxic for RGCs, and hWJMSC isolated from different umbilical cords delay axotomy-induced RGC loss. (**A**) Column graph showing the total number of RGCs ± standard deviation (SD) in retinas analyzed 7 days after administration of increasing doses of hWJMSC (10,000, 20,000 and 40,000). In these retinas, the total number of RGCs did not differ among them nor with that found in intact retinas (n.s. *p* > 0.05. ANOVA, Tukey’s post hoc test). n = 4 retinas/dose. (**B**) Column graph showing the total number of surviving RGCs ± SD one week after ONC and treatment with vehicle (n = 4 retinas), or increasing doses of hWJMSC (n = 4 retinas/dose). In the retinas treated with hWJMSC, the number of RGCs was significantly higher than in the vehicle group (***p < 0.001, ANOVA, Tukey’s post hoc test) and the neuroprotection elicited was similar for all hWJMSC doses (n.s. *p* > 0.05). (**C**) Scatter graph (total number of RGCs ± SD *vs*. days post-axotomy) showing the course of RGC loss in ONC-injured retinas treated with vehicle (n = 4–6/time point), or with 20,000 hWJMSC isolated from three different umbilical cords (UC1, UC2, UC3, n = 4–7/time point and UC). RGC survival was similar among hWJMSC-treated retinas both between cells isolated from the same UC and among UC (*p* > 0.05, ANOVA, Tukey’s post-hoc test). At 7 and 14 days post-ONC, the number of surviving RGCs was significantly higher in all hWJMSC-treated retinas compared to vehicle ones (***p < 0.001, ANOVA; Tukey’s post-hoc test). Thirty days after the injury, the number of RGCs was similar in all groups (n.s. *p* > 0.05). (**D**) Column graph showing the percent of surviving RGCs ± SD in hWJMSC-treated retinas (data from all UC pooled) compared to vehicle-treated ones at the same time points (100%). The higher percent of neuroprotection was found at 14 days post-lesion (**p < 0.01; ***p < 0.001. ANOVA, Tukey’s post-hoc test).

To assess the possible toxicity of different doses of hWJMSCs injected intravitreally on RGCs, we injected either vehicle or 10,000, 20,000 or 40,000 cells into the vitreous chamber of otherwise intact retinas, and 7 days later we quantified the total number of RGCs (n = 4/dose). As shown in Fig. 2A, and compared to vehicle-injected retinas none of the hWJMSCs doses decreased significantly the population of RGCs.

### hWJMSCs are neuroprotective for RGCs

Next, we wondered whether hWJMSCs exerted a neuroprotective effect on axotomized RGCs and if so, whether it was dose-dependent. The same doses as before were injected right after ONC (n = 4/dose), and retinas analyzed 7 days later when without treatment more than half of the RGCs have died^18,21,39,40^. In Fig. 2B, is observed that in all hWJMSCs-treated retinas there were significantly more surviving RGCs than in the vehicle-treated ones (p < 0.001). Furthermore, all doses seemed to be similarly neuroprotective. Thus, for subsequent experiments we chose the medium dose, 20,000 cells/injection.

### hWJMSC from different umbilical cords delay axotomy-induced RGC death

To investigate whether hWJMSCs isolated from different umbilical cords produced the same neuroprotection or if it was cordon-specific, as well as to know for how long this neuroprotection lasted, hWJMSCs isolated from 3 different umbilical cords were administered intravitreally right after ONC (n = 4–7 retinas/time point and umbilical cord) and the retinas analyzed 7, 14 or 30 days later. Quantitative data in Fig. 2C show that the number of RGCs in the transplanted retinas was similar among umbilical cords at all time points analyzed, and significantly higher than in vehicle-treated retinas at 7 and 14 days after the lesion (p < 0.001). Averaging the data of all the hWJMSCs-treated retinas the mean number of RGCs ± standard deviation at 7, 14 and 30 days was 61,773 ± 9371; 34,338 ± 4254 and 7291 ± 1379, respectively, while in vehicle-treated retinas was 34,549 ± 9168; 11,772 ± 3535 and 7810 ± 2840 at the same time points. In percent, this increased survival amounts to 179 ± 21 and 291 ± 36 at 7 and 14 days post-lesion, respectively (p < 0.001) and to 96 ± 19 at 30 days (Fig. 2D).

**Figure 3:**
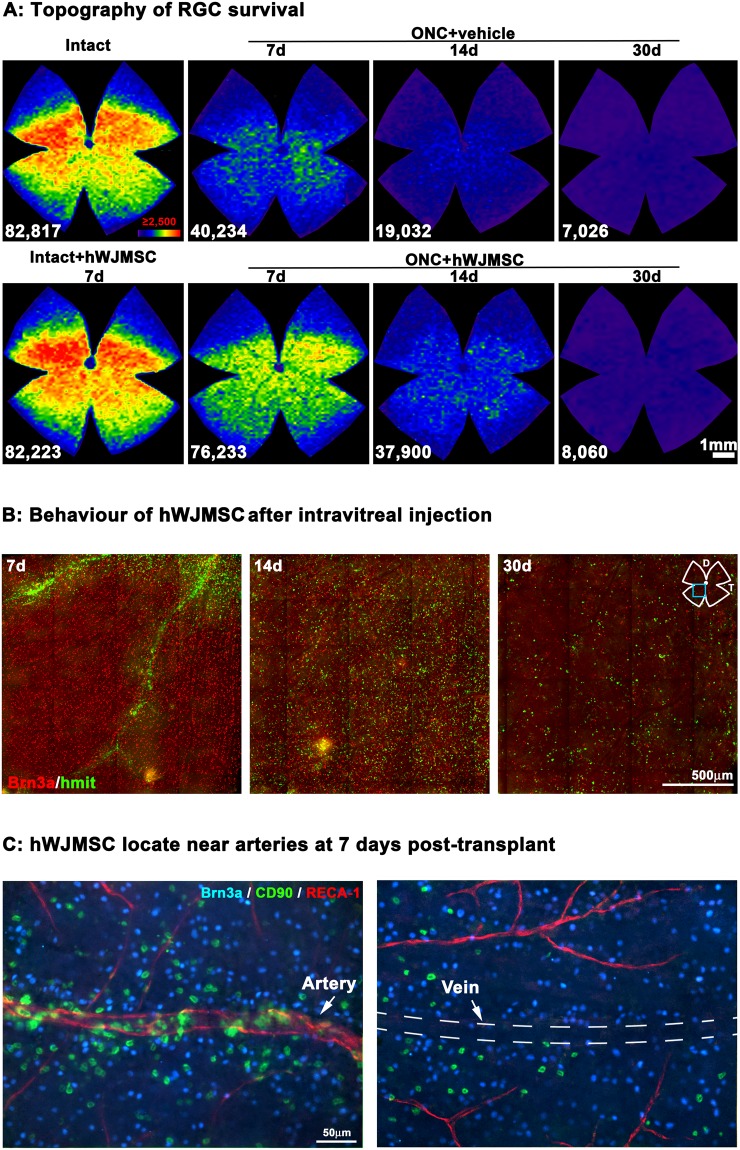
Topography of RGC survival and hWJMSC long-term survival after intravitreal administration. (**A**) Isodensity maps showing the distribution of RGCs in intact retinas, in intact retinas analyzed 7 days after hWJMSC administration, and in ONC+hWJMCs or ONC+vehicle retinas analyzed at 7, 14 or 30 days. Density colour scale (at the bottom right of the top left panel) goes from 0 (purple) to ≥2,500 RGCs/mm^2^ (red). At the bottom of each map is shown the number of RGCs counted in the original retina. (**B**) Double immunodetection of Brn3a^+^RGCs and hmit^+^hWJMSC in flat mounted ONC+hWJMSC retinas analyzed at 7, 14 or 30 days. The blue square in the retinal drawing marks the retinal region where these images were taken from. (**C**) magnifications taken from a flat mounted ONC+hWJMSC retina analyzed at 7 days showing Brn3a^+^ RGCs (blue), CD90^+^ hWJMSC (green) and RECA1^+^ arteries (red). The location of a vein is marked with the dashed line. (**D**) dorsal, T: temporal.

Topographically (Fig. 3A), RGC neuroprotection by hWJMSCs occurrs across the retina, with no sectors of survival, whether the neuroprotection is mediated by cell-contact and/or by a paracrine mechanism.

### Intravitreally transplanted hWJMSCs integrate in the ganglion cell layer and last up to 30 days

Seeing the pattern of RGC survival, it was important to know for how long hWJMSCs survived and/or whether they were able to integrate in the ganglion cell layer. Seven days after the injection, hWJMSCs were found in the central retina, spreading along the inner retinal arteries (Fig. 3B,C). By day 14, they had extended all across the retina, and by day 30 there were fewer although still abundant (Fig. 3B). It is worth noting that RGCs and hWJMSCs were photographed without changing the focus, indicating that hWJMSCs integrate in the ganglion cell layer (GCL, see below).

### hWJMSCs transiently over-express anti-inflammatory cytokines and trophic factors after intravitreal administration, preferentially when transplanted into injured retinas

**Figure 4:**
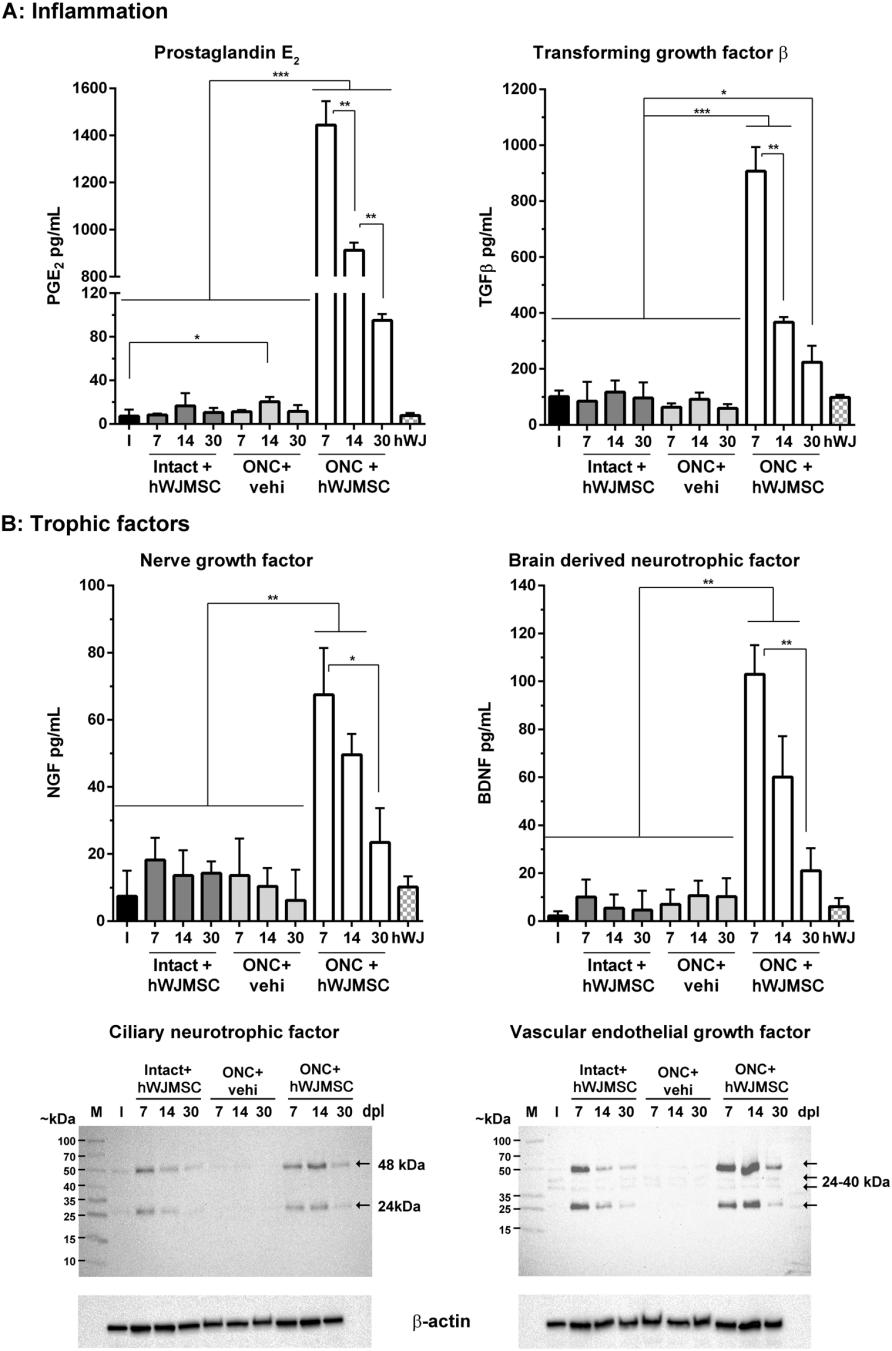
hWJMSC over-express cytokines and trophic factors after intravitreal administration. (**A**) Graph bars from ELISAs assays showing the concentration ± SD (pg/mL) of PGE_2_ (left) and TGFβ (right) in retinal extracts from intact retinas (I) and intact+hWJMSC, ONC+vehicle, ONC+hWJMSC dissected at 7, 14 or 30 days after cell administration and/or ONC. The last column corresponds to extracts from primary cultures of hWJMSC (hWJ). (**B**) Top row: graph bars from ELISAs assays showing the mean concentration ± SD (pg/mL) of NGF and BDNF. Bottom row, western blotting of CNTF and VEGF in the same extracts as above (hWJMSC extracts were not used in the western blots). The expression levels of these proteins were higher in injured retinas treated with hWJMSC compared to intact, intact+hWJMSC or ONC+vehicle. Note that all these assays were done with human-specific antibodies, although species cross-reactivity exists, mostly for PGE_2_^41^. Extracts are pools from n = 4 retinas/time point and group. *p < 0.05; **p < 0.01; ***p < 0.001, ANOVA Tukey’s post-hoc test.

First, we analyzed whether once transplanted, hWJMSCs expressed PGE_2_, TGFβ, and IDO as they did in the mixed cultures shown above. ELISA data in Fig. 4A show that the levels of both, PGE_2_ and TGFβ, were highly increased in extracts from injured and transplanted retinas compared to intact, intact+hWJMSC and ONC+vehicle retinal extracts. Importantly, both PGE_2_ and TGFβ levels were highest at 7 days post-transplantation (p < 0.001), decreasing gradually thereafter (p < 0.01). Of note, although both ELISAs are human specific, PGE_2_ has been reported to work on rat’s extracts^41^. Nevertheless the concentration of PGE_2_ found in retinal extracts without hWJMSC was ~100 times lower than in transplanted ones. Finally, contrary to the *in vitro* results, human IDO was not detected in the transplanted retinas (not shown).

Then, we measured the levels of several trophic factors known to be neuroprotective for axotomized RGCs. NGF and BDNF levels were quantified by ELISA, and CNTF and VEGF by western blotting. In all cases we used human-specific antibodies. As shown in Fig. 4B, NGF and BDNF levels increase significantly in injured and transplanted retinas compared to the control extracts (p < 0.01). Again, their levels peak at 7 days, declining thereafter (p < 0.01, p < 0.05). Regarding CNTF and VEGF, their levels increase in hWJMSCs-transplanted retinas, both intact and injured, compared to non-transplanted ones, where they are almost undetectable. In intact+hWJMSC extracts, CNTF and VEGF levels were highest at 7 days decreasing thereafter, while in ONC+hWJMSC samples, they were highest at 14 days declining at 30 days.

### hWJMSC transplant disrupts the retinal architecture due to a massive infiltration of macrophages/microglial cells

**Figure 5:**
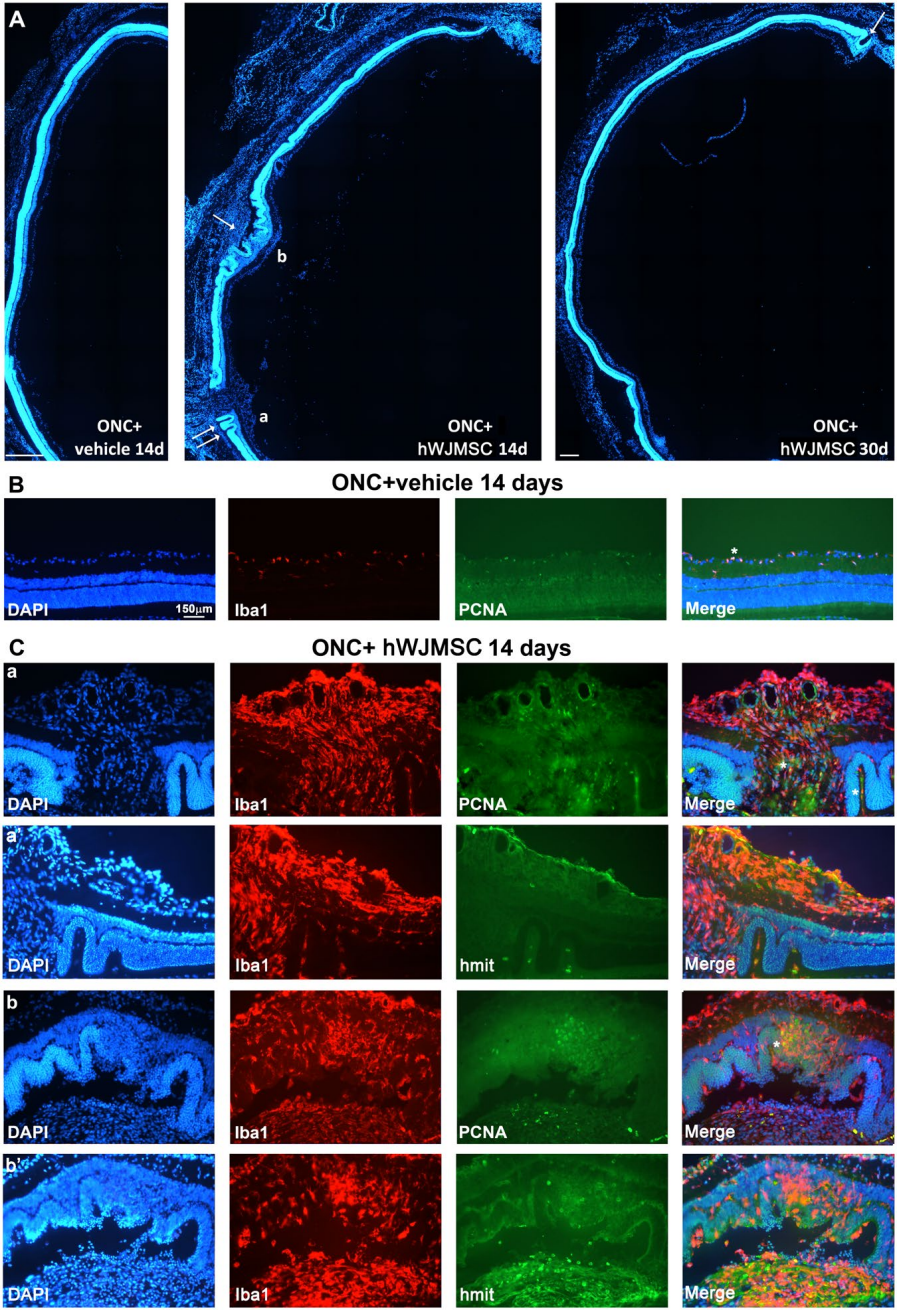
Intravitreal transplant of hWJMSC into rat retinas induces a severe microglial reaction and retinal restructuration. (**A**) DAPI-stained photomontages from cross sections of eyes dissected 14 days after ONC+vehicle or 14 and 30 days after ONC+hWJMSC intravitreal transplant. The dorsal retina is at the top of the image. In the hWJMSC groups, there are abnormal folds going from the outer to the inner retina (arrows). (**B**) Magnification from the ONC+vehicle retinal section showing Iba1 (microglial cells) and PCNA (dividing cells) staining. Some of these microglial cells have divided (asterisks). Microglial division was not observed in intact retinas (not shown). (**C**) magnifications from the ONC+hWJMSC 14 days retinal section in A. These images were taken from the areas marked a,b and show Iba1 and PCNA immunodetection. a’,b’ are magnifications from consecutive sections acquired in the same areas as a,b and immunodetected with Iba1 and h-mit (hWJMSC). Scale bars in A, 500 µm.

Finally, we performed an anatomical study on retinal cross-sections. In Fig. 5A is observed that in ONC+hWJMSC retinas, but not in ONC+vehicle ones, the outer retina was folded and separated from the choroid (retinal detachment, arrows) breaking the retinal layered structure. Furthermore, these folds were observed in all the transplanted retinas, included intact ones (not shown), and were more frequent at 14 than at 30 days. In these sections, we immunodetected macrophages/microglial cells (Iba1) and hWJMSCs (h-mitochondria) together with a marker of cell proliferation (PCNA). In ONC+vehicle retinas, microglial cells were observed in the ganglion cell layer and both plexiform layers and some of them were PCNA^+^ (Fig. 5B). In intact retinas, no dividing microglial cells were observed (not shown). By contrast, in the transplanted retinas (Fig. 5C) Iba1^+^cells, displaying the typical reactive hypertrophic phenotype, were observed entering the retina from the choroid plexus and causing the retinal grooves. Also, these invading macrophages underwent division. In some cases, hWJMSCs were seen in the outer retina (Fig. 5C,b’) but they were mostly located in the inner retina, as abovementioned (Fig. 5C,a’) and around blood vessels, where microglial cells were also concentrated.

## Discussion

In this work we have demonstrated that hWJMSCs isolated from three different human umbilical cords and transplanted into the rat vitreous body are not toxic for RGCs, and neuroprotect axotomized RGCs during the quick phase of death, up to 14 days. Thereafter RGC loss equals that observed in vehicle-treated retinas. Interestingly, the decline of neuroprotection coincides with fewer hWJMSCs present in the retina and a decrease of secreted human trophic factors whose levels, unexpectedly, differ when the transplanted retina is intact or injured. Finally, we have observed that hWJMSCs induce a massive migration of microglial/macrophages from the choroid to the inner retina that disrupts the retinal architecture

hWJMSCs are non-immunogenic since a single injection of MHC-mismatched unactivated umbilical cord tissue-derived cells does not induce a detectable immune response. However, when hWJMSCs are injected into an inflamed region, repeatedly injected in the same area or stimulated with IFN-γ before injection, they are immunogenic^42,43^. The finding that MSC induce inhibition of T cells has clinical implications since allogeneic stem cell transplantation could modulate the immune response during stem cell-based regenerative therapies. Several studies have shown that extra-embryonic MSC significantly express a higher level of immunomodulatory factors (e.g. TGFβ1, IDO, PGE_2_) in comparison to adult bone marrow-derived MSCs^44^. This fact matches well with what we found after performing the *in vitro* characterization of the immunological properties of the hWJMSCs. Here we show that hWJMSCs: i/do not induce proliferation of allogeneic T cells; ii/suppress the proliferation of T cells induced by allogeneic mDCs cells; iii/secrete soluble factors that mimic the immunosuppressive effects associated with the co-culture of the MSCs with the T cells (i.e. TGFβ, IDO, and PGE_2_), and iv/inhibit the production of pro-inflammatory cytokines (e.g. IFN-γ) of T cells stimulated by an allogeneic stimuli. Importantly, our data are consistent with previously reported results that showed that hWJMSCs exhibit more potent immunomodulatory properties than adult bone marrow MSCs^7^.

*In vivo*, hWJMSCs integrate into the ganglion cell layer and survive up to 30 days. They first locate along the retinal arteries vessels, a behavior that has been also observed after intracerebral grafting^45^. By day 14 they cover the retina and it is at this time point when they reach their peak. During this period it is possible that they divide, though we did not observe tumors in agreement with previous works in the retina^46^ or the brain^45,47^. Thirty days after the transplant their number has greatly decreased. Although the survival found here is in accordance with other works in brain^45,48^, it is important to have in mind that because this is a xenograft, hWJMSCs decrease at 30 days may be caused by tissue rejection and clearance^49,50^. In fact, the massive macrophage/microglia activation observed in the transplanted retinas advocates for this possibility.

In intact retinas, hWJMSC transplant does not cause RGC death. This is, to our knowledge, the first work assessing the toxicity of UC-derived cells on RGCs. This is an important assay firstly, because safety experiments should be done in animal models if these, or other cells, are going to be translated into the clinic. Secondly, because the neuroprotection elicited by UC-derived MSCs in models of RGC axonal damage is often very small (hWJMSCs, 22% higher than no treatment after ocular hypertension^35^; UC-MSC, 28% after ONT^51^) and this could be because the transplanted cells exert a direct or indirect (through glial cells) toxic effect that interferes with the neuroprotection.

In line with this, we show that while the graft does not kill RGCs, it is not completely harmless, as the retinal architecture is damaged by the infiltration and activation of Iba1^+^cells. This response is not surprising since this is a xenotransplant albeit of cells with limited immunogenicity^3^ and into an immuno-privileged environment. Nevertheless, this activation has been observed as well in allograft transplants into the mouse retina^52^. It is important to have in mind that the infiltration of Iba1^+^cells was transient, decreasing at 30 days post-transplant when the number of hWJMSCs in the retina had also diminished. Importantly retinal architecture at 30 days was better than at earlier time points, indicating that once the hWJMSCs have disappeared, the system can be restored.

Iba1^+^ cells might be either microglial cells, the resident macrophages of the CNS, or invading macrophages which upon entering the CNS parenchyma are not distinguishable from microglial cells. Here, those Iba1^+^cells located in the inner retina are most probably resident microglial cells, while those in the outer retina would be a mixture of both populations. Nonetheless, these cells have the morphological attributes of activation. To what extent and whether the excess of activated Iba1^+^ cells impairs retinal function or affects the observed neuroprotection in injured retinas we do not know. The role of microglial cells in neurodegeneration is controversial^53^, and it has been reported that MSC modulate them towards a restorative phenotype^5^. Although Giunti *et al*.^5^ used allografts and the experiments here are xenografts, the same modulation may happen and thus, the excess of Iba1^+^ cells may not be harmful to neurons, being their role a cleansing one. Furthermore, part of the neuroprotection observed here may be due to them being in a restorative state. Although the infiltration of these Iba1^+^ cells might be caused by tissue rejection as above-mentioned, we cannot forget that MSC express macrophage attracting-chemokines^54^. Finally, we did not investigate astrocytes and Müller cells, but these glial cells are activated by topical treatment^41^, intravitreal injections^55^, RGC death^56^, and MSC grafts^52^.

We show that hWJMSCs isolated from three different umbilical cords elicit the same RGC neuroprotection. This is a promising result for translational medicine because due to the high genetic variability among human donors, each cord may have had different properties.

We observed RGC neuroprotection at 7 and 14 days (179 and 291% higher compared to untreated retinas, respectively). This is, to our knowledge, the higher neuroprotection reported in *in vivo* models of RGC axonal damage treated with MSC derived from the bone-marrow (160% higher than no treatment at 14 days after ONT^27^), UC-blood (28% after ONT^51^), or WJ (22% after ocular hypertension^35^). However, these percentages may not be fully comparable because of the different axonal injuries, cellular doses used in each work, and RGC quantification methods (sampling *vs*. whole population). Nevertheless, there are two common denominators among these works and ours: RGC survival is transitory, and the transplanted cells secrete neuroprotective trophic factors.

In fact, the higher RGC survival by hWJMSC transplant, may be explained alone by the higher levels of trophic factors found in the transplanted retinas^20–22,24–26^. Interestingly, our data show that the higher levels of BDNF, NGF, CNTF and VEGF are not better neuroprotecting RGCs than a single treatment with BDNF or CNTF alone^20–22^, suggesting that the effect of these factors is not additive, at least at the levels expressed in the transplanted retinas.

The loss of neuroprotection coincides not only with fewer hWJMSCs in the retina, and consequently with a lower level of released PGE_2_, TGFβ and neurotrophic factors, but also with the second, slow phase of axotomy-induced RGC death^16–18,40^. With the exception of knock-out strains lacking key pro-apoptotic genes^57^, most neuroprotective treatments after optic nerve axotomy last no longer than 14–21 days^20–22,24–26^, even when prolonged delivery of BDNF, the best neuroprotectant, is achieved^58^. It has been postulated that this happens because RGCs are no longer receptive to the treatment. We propose here that it could be also related to their mode of death. During the first two weeks most of the RGCs die by apoptosis^16,59^, and during this phase, microglial cells do not have a role in their death^40,60^. During the second slow phase, the mechanism of RGC death has not been described, but hypothetically it may relate to an altered microglial activation. PGE_2_ increases in axotomized retinas with or without transplant as shown before^41^ and here, and TGFβ increases in axotomized and transplanted retinas. Both soluble factors are immunomodulators with a described anti-inflammatory function^61–63^ and PGE_2_ may also be neuroprotectant^64–66^. When these two mediators decrease, the retinal inflammatory environment may change to neurotoxic, by altering the microglial phenotype and causing the second wave of RGC loss.

It has been suggested that depending on the pathophysiological microenvironment MSC release different cytokines and trophic factors^47,67–69^. We show here that the secretion of TGFβ, PGE_2_, NGF, BDNF, VEGF and CNTF in intact and transplanted retinas is much lower than in axotomized and transplanted ones. Because we used human-specific ELISAs or antibodies (although some cross-reactivity may occur), these factors are mostly secreted by the hWJMSC. Thus, it becomes clear that injured neurons emit signals that directly or indirectly (through glial cells) stimulate hWJMSC to a given secretome. It is, therefore, tempting to speculate that hWJMSC produce bespoken secretomes to accommodate the needs of the injured tissue.

In summary, hWJMSCs transplanted into the axotomized rat retina are neuroprotective most possibly due, but not limited to, their release of anti-inflammatory and neurotrophic factors. We also demonstrate that this xenotransplant is not innocuous, as it attracts Iba^+^ cells that invade the retina disrupting its architecture. Whether this infiltration has a role in the elicited neuroprotection is not known but it would be of interest to investigate the rescue of RGCs and the inflammatory response in immunosuppressed animals or in syngenic transplants, since allografts seem to cause the same response^52^. Finally, the secretome of hWJMSCs changes depending on the retinal state, widening the potential therapeutic use of these cells for a variety of neurodegenerative diseases.

## Material and Methods

### Animal Handling and Ethics Statement

Female albino Sprague Dawley rats (2 months old) were obtained from the University of Murcia (Murcia, Spain) breeding colony at the animal housing facilities and were housed with a 12:12 hours (h) light:dark cycle (maximum 50 luxes). Animal care and experimental procedures were performed in accordance to the Association for Research in Vision and Ophthalmology, European Union guidelines for the use of animals in research and were approved by the Ethical and Animal Studies Committee of the University of Murcia (248/2016).

Animals undergoing surgery were anesthetized by intraperitoneal injection of a mixture of ketamine (60 mg/kg; Ketolar, Pfizer, Alcobendas, Madrid, Spain) and xylazine (10 mg/kg; Rompun, Bayer, Kiel, Germany). Analgesia was provided by simultaneous administration of buprenorphine (0.1 mg/kg; Buprex, Buprenorphine 0.3 mg/mL; Schering-Plough, Madrid, Spain). During and after surgery, the eyes were covered with an ointment (Tobrex; Alcon S. A., Barcelona, Spain) to prevent corneal desiccation. Euthanasia was carried out by an intraperitoneal injection of an overdose of sodium pentobarbital (Dolethal, Vetoquinol; Especialidades Veterinarias, S.A., Alcobendas, Madrid, Spain).

Animal groups: i/intact+ intravitreal administration of hWJMSC (n = 4 retinas/dose); ii/optic nerve crush + intravitreal injection of vehicle (n = 4–7 retinas/time point); iii/optic nerve crush + intravitreal injection of hWJMSC (n = 4–7 retinas/dose, umbilical cord and time point). Retinas were dissected at 7, 14 or 30 days after cell administration. Intact retinas were used as controls.

### Isolation of Wharton’s jelly mesenchymal stem cells from human umbilical cords

Three different human umbilical cords were collected from patients undergoing full-term pregnancy elective for caesarean section. Tissue collection was approved by the biobank of the Instituto Murciano de Investigación Biosanitaria-Virgen de la Arrixaca, (IMIB-Arrixaca Murcia, Spain). Donors provided an informed written consent which was approved by and followed the guidelines of the Ethics Committee the Hospital Clínico Universitario Virgen de la Arrixaca (Murcia, Spain).

Human Wharton’s jelly mesenchymal stem cells (hWJMSC) were isolated by the explant method according to previously described protocols^70,71^. Briefly, each umbilical cord was sectioned into 3–5-cm long pieces, amnion was cut along the horizontal axis, and blood vessels with clots inside were removed. Then, cord pieces were placed with the inside facing to the bottom of a sterile 10-cm^2^ petri dish. Explants were left to attach to the plate and complete culture medium (DMEM medium supplemented with 15% (v/v) fetal bovine serum (FBS), 1% (v/v) L-glutamine and 1% (v/v) penicillin/streptomycin, all from Life Technologies, Carlsbad, CA, USA) was added. The medium was replaced every three days and the hWJMSC attached to the plate were grown up to 80–90% confluence before doing serial passages.

### Characterization of hWJMSC

Human WJMSC were analyzed for the positive expression of the mesenchymal stem cell markers CD73, CD90 and CD105, and negative expression of the hematopoietic markers CD14, CD20, CD34 and CD45 by flow cytometry (all antibodies for Miltenyi Biotec, Bergisch Gladbach, Germany), following the guidelines of the International Society for Cellular Therapy to confirm mesenchymal phenotype^72,73^. In addition, the expression of the co-stimulatory molecules CD80 and CD86 (Biolegend, San Diego, CA, USA) or major histocompatibility complex class II (HLA-DR) (Biolegend) was analyzed. Flow cytometry experiments were performed with a BD FACS Canto II flow cytometer (BD Biosciences, San Diego, CA, USA) and further analyzed with Kaluza analysis software (Beckman Coulter, Inc., Brea, CA, USA). Human WJMSC were tested as well for their capacity to differentiate to adipocytes, chondroblasts, and osteoblasts as previously described^74,75^.

### Viability of isolated hWJMSC in different vehicles

To avoid administration of dead cells it was important to assess the temporal viability of isolated hWJMSC. Cells were re-suspended in either phosphate buffer saline (PBS) or Dulbecco’s Modified Eagle’s Medium (DMEM) and kept at 4 °C. Cell viability was measured by trypan blue exclusion and automatic quantification (Coulter Z2, Beckman Coulter Life Sciences, USA) every 15 minutes (min) up to 180 min. Human WJMSC viability in PBS ranged between 80 and 85% up to 150 min and then halved at 165 min, while in DMEM cell viability was higher than 85% up to 180 min. Therefore, we chose DMEM as isolation and transplantation vehicle.

### Optic nerve crush (ONC) and intravitreal injection

The left optic nerve was crushed at 3 mm from the optic disc, following previously described methods^18,20^. In intact animals or immediately after the axotomy, cells or vehicle were injected into the vitreous chamber of the left eye using standard procedures in our group^20–22^.

### Tissue preparation and sectioning

Unless otherwise stated, all reagents were from Sigma-Aldrich Quimica S.A. Madrid, Spain.

For anatomical analyses, all animals were perfused transcardially with 0.9% saline solution followed by 4% paraformaldehyde in 0.1 M phosphate buffer.

For RGC quantification and identification of hWJMSC, retinas (n = 4–7/group and time point) were dissected as flattened whole-mounts as previously described^39^. Some eye cups (n = 3/group and time point) were cryoprotected in a series of sucrose gradients, embedded in optimal cutting temperature medium (OCT, Tissue-Tek, Sakura-Finetek, VWR, Barcelona, Spain) maintaining their orientation (the dorsal rectum muscle was kept as mark), frozen, and sectioned in a cryostat at 14 µm thickness.

### Immunodetection

Immunodetection in flat mounts or cross-sections was carried out as previously described^39,76^. RGCs were detected using goat α-Brn3a (C-20; Santa-Cruz Biotechnology, Heidelberg, Germany) diluted 1:750. Microglial cells were identified using rabbit α-Iba1 antibody (ionized calcium-binding adapter molecule 1; ab-178846 Abcam, Cambridge, UK) diluted 1:500. hWJMSC were detected with mouse α-human mitochondria diluted 1:800 (ab92824, Abcam, Cambridge, UK) or rabbit α-CD90 (1:200). Proliferating cells were identified with mouse α-PCNA (Proliferating Cell Nuclear Antigen; sc-56, Santa Cruz Biotechnology,) diluted 1:50. Rat arteries with mouse α-RECA1 (1:50; rat endothelial cell antigen 1; MCA970GA AbDSerotec/Bio-Rad, Bionova Científica, Madrid, Spain).

Secondary detection was carried out with donkey α-goat, donkey α-rabbit or donkey α-mouse coupled to Alexa Fluor 594, 488 or 355 (Molecular Probes; Thermo Fisher Scientific, Madrid, Spain) diluted at 1:500.

In retinal sections, all nuclei were counterstained using antifading mounting medium with 4′,6-diamidino-2-phenylindole (DAPI, Vectashield mounting medium with DAPI; Vector Laboratories, Palex Medical, Barcelona, Spain).

### Image acquisition and analysis

All images were acquired using an epifluorescence microscope (Axioscop 2 Plus; Zeiss Mikroskopie, Jena, Germany) equipped with a computer-driven motorized stage (ProScan H128 Series; Prior Scientific Instruments, Cambridge, UK) controlled by image analysis software (Image-Pro Plus, IPP 5.1 for Windows; Media Cybernetics, Silver Spring, MD, USA). Retinal photomontages (flat mounts or cross-sections) were reconstructed from 168 (12 × 14) individual 10x images by zig-zag tiling, as reported^39^. Individual images were acquired with a 20x objective.

The whole population of Brn3a^+^RGCs was quantified automatically and their distribution assessed by isodensity maps using previously reported methods^39^. Maps were plotted using SigmaPlot (SigmaPlot 9.0 for Windows; Systat Software, Inc., Richmond, CA, USA).

### Immunomodulatory properties of hWJMSC

Mixed lymphocyte culture (MLC) experiments were used to evaluate hWJMSC ability to suppress T cell proliferation after stimulation with allogeneic mature dendritic cells. Firstly, monocytes were isolated from peripheral blood samples from healthy volunteers using the Human Monocyte Enrichment Cocktail (StemCell Technologies, Grenoble, France). Then, for immature myeloid dendritic cells (iDCs) differentiation, 50 ng/ml granulocyte-macrophage colony-stimulating factor (GM-CSF) (Sigma-Aldrich, St. Louis, MO, USA) and 25 ng/ml interleukin-4 (IL-4) (Invitrogen, Waltham, MA, USA) were added to the culture medium every three days for seven days. By day seven, more than 95% of cells were CD14^−^ CD11c^+^ CD1a^+^ BDCA-1^+^ (not shown). Finally, mature myeloid dendritic cells (mDCs) were obtained from iDCs by stimulation with 200 ng/ml lipopolysaccharide (LPS; Sigma-Aldrich) for additional 24 hours as reported^77^. On the other hand, human T cells were purified from peripheral blood mononuclear cells using Pan T cell isolation kit (Miltenyi Biotec). Then, the MLC was performed by incubating responders T cells and allogeneic stimulator mDCs at a 5:1 ratio, in 96-well round bottom plates (Sarstedt, Nümbrecht, Germany) for five days. Human WJMSC were previously seeded at several ratios (MSCs: T cell) in triplicates, to study the dose-response suppressive effect of the hWJMSC on the proliferation capacity of T cells. Human WJMSC and mDCs were previously irradiated with 30 Gy after being seeded onto the plates to prevent their proliferation. In some experiments, the inhibitors indomethacin (50 µM), 1-methyl-DL-tryptophan (1 µM), and SB-431542 (10 µM)were added from the beginning of the MLC and replaced in the cultures every two days. After, cell proliferation was determined using an enzyme-linked immunoabsorbent assay (ELISA) BrdU colorimetric kit (Roche Diagnostic, Mannheim, Germany) according to the manufacturer’s instructions.

Supernatants of MLC were harvested on day five before the addition of BrdU to quantify released cytokines. ELISA kits for human transforming growth factor-β (TGFβ, eBioscience, Hatfield, United Kingdom), prostaglandin E_2_ (PGE_2_, R&D Systems, Minneapolis, MN, USA), indoleamine 2,3-dioxygenase (IDO, Cusabio Biotech, BioNova Cientifica, Madrid, Spain), and interferon γ (INF-γ, Diaclone, BioNova Cientifica,) were used to measure the levels of these molecules following the manufacturer’s protocols.

### Western blotting and ELISAs from retinal extracts

Fresh dissected retinas (n = 4/group and time point) were homogenized in lysis buffer (Pro-prep protein extraction solution; Intron Biotechnologies, Sevilla, Spain). Protein concentration was determined using SimpliNano spectrophotometer (Biochrom Ltd, Cambridge, UK). For western blotting, a total of 10 to 40 µg of protein (pool from the 4 retinal extracts) were resolved in 4% to 20% SDS-PAGE gels (Bio-Rad laboratories, SA, Madrid, Spain) and transferred to nitrocellulose membranes (GE Healthcare, Barcelona, Spain) by electroblotting. Blots were blocked for 1 hour with 5% skim milk in PBS containing 0.5% Tween-20 and then were incubated overnight at 4 °C with mouse α-human VEGF (1:2000, vascular endothelial growth factor, ab2109, Abcam, Cambridge, UK) or mouse α-human CNTF (1:500 ciliary neurotrophic factor, ab17284, Abcam, Cambridge, UK). Secondary detection was carried out with horseradish peroxidase (HRP)-conjugated α-mouse antibody (Santa Cruz Biotechnologies) at 1:5000 dilution. Membranes were developed using ECL Prime Western Blotting Detection Reagent (GE Healthcare). As loading control, total protein/lane in the membranes was visualized using reversible protein staining (R-PROB, Sigma-Aldrich) followed by β-actin identification using anti-βactin–HRP mouse monoclonal antibody (Sigma-Aldrich). β-actin signal was equivalent to total protein staining in all lanes, and thus its intensity was used for normalization. The density of the protein bands was acquired using image analyzer Chemidoc XRSþ and ImageLab 5.2.1 software (Bio-Rad, Hercules, CA, USA).

ELISA assays were carried out on the same retinal lysates used for western blotting (triplicates of 10 µg of total protein) and/or in hWJMSC extracts, homogenized in the same lysis buffer as above, following the manufacturer’s instructions to measure human TGFβ1 (eBioscience,), human PGE_2_ (R&D Systems, Minneapolis, MN, USA), human IDO (Cusabio Biotech,), human BDNF(Promega, Madison, WI, USA), human NGF)(Elabscience, Bethesda, MD, USA) and human IFN-γ (Diaclone).

### Statistical Analysis

Data were analyzed with GraphPad Prism v.7 (GraphPad San Diego, USA). RGC survival, BrdU, and ELISAs experiments were analyzed between each group and among groups (one-way ANOVA, post hoc test Tukey’s test). Differences were considered significant when p < 0.05. Data are presented as mean ± standard deviation.

## Electronic supplementary material

Supplementary figures
