# Supplementary material for: Human Wharton’s jelly mesenchymal stem cells protect axotomized rat retinal ganglion cells via secretion of anti-inflammatory and neurotrophic factors
Source: Sci Rep. 2018 Nov 2;8:16299. doi: 10.1038/s41598-018-34527-z (PMC6214908; doi:10.1038/s41598-018-34527-z)
Supplement: Supplementary file 1 — Supplementary figures [file 41598_2018_34527_MOESM1_ESM.pdf]

Human Wharton's jelly mesenchymal stem cells protect axotomized rat retinal ganglion cells via secretion of anti-inflammatory and neurotrophic factors

Jose E. Millán-Rivero[1,2#], Francisco M. Nadal-Nicolás[3,4§#,] David García-Bernal[1,2], Paloma Sobrado-Calvo[3,4], Miguel Blanquer[1,2], Jose M. Moraleda[1,2], Manuel Vidal-Sanz[3,4], Marta Agudo-Barriuso[3,4]*

[1]Unidad de Terapia Celular y Trasplante Hematopoyético. Instituto Murciano de Investigación Biosanitaria Virgen de la Arrixaca (IMIB-Arrixaca). Murcia. Spain. [2]Dpto Medicina Interna, Universidad de Murcia, Murcia, Spain. [3]Dpto Oftalmología, Universidad de Murcia, Murcia, Spain. [4]Grupo de Oftalmología Experimental, Instituto Murciano de Investigación Biosanitaria Virgen de la Arrixaca (IMIB-Arrixaca). Murcia. Spain.

[§]Current address: Retinal Neurophysiology Section, National Eye Institute, National Institutes of Health, Bethesda, MD, USA.

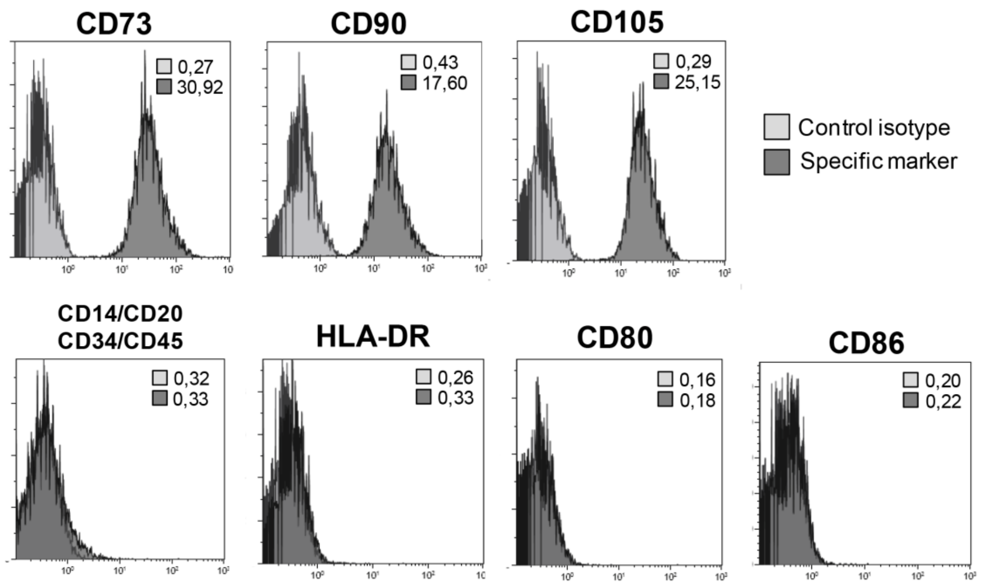

Supplementary Figure 1. Characterization of human Wharton's jelly mesenchymal stem cells. Mesenchymal stem cells isolated from the Wharton's jelly of the human umbilical cord express characteristic MSCs markers such as CD73, CD90, and CD105, whereas expression of hematopoietic/co-stimulatory markers CD14, CD20, CD34, CD45, HLA-DR, CD80, and CD86 are low or negative. Insert numbers represent mean fluorescence intensity values from viable cells. Histograms show representative flow cytometry results obtained from three independent experiments.

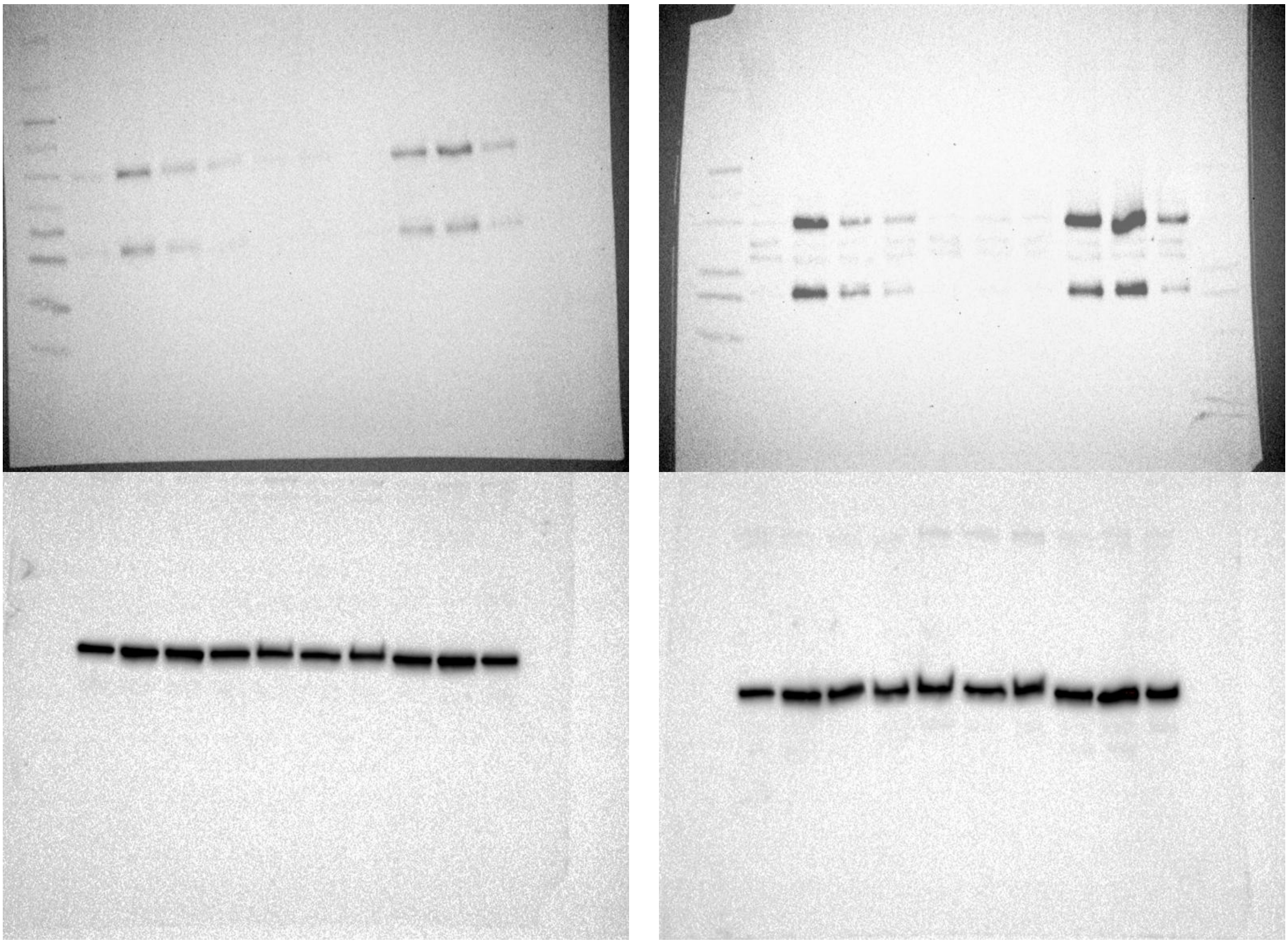

Supplementary figure 2: Original full-length western blots
Top left: CNTF
Top right: VEGF
Bottom: β-actin
